# The role of CPT1A as a biomarker of breast cancer progression: a bioinformatic approach

**DOI:** 10.1038/s41598-022-20585-x

**Published:** 2022-09-30

**Authors:** Mitali Das, Athina Giannoudis, Vijay Sharma

**Affiliations:** 1 Department of Molecular and Clinical Cancer Medicine, Institute of Systems, Molecular and Integrative Biology, University of Liverpool, Liverpool, UK; 2 Liverpool Clinical Laboratories, Department of Cellular Pathology, Royal Liverpool University Hospital NHS FT, Liverpool, UK

**Keywords:** Molecular biology, Diagnostic markers, Predictive markers, Prognostic markers, Breast cancer, Mechanisms of disease, Clinical genetics, Fatty acids

## Abstract

Breast cancer is the commonest malignancy of women and with its incidence on the rise, the need to identify new targets for treatment is imperative. There is a growing interest in the role of lipid metabolism in cancer. Carnitine palmitoyl-transferase-1 (CPT-1); the rate limiting step in fatty acid oxidation, has been shown to be overexpressed in a range of tumours. There are three isoforms of CPT-1; A, B and C. It is CPT-1A that has been shown to be the predominant isoform which is overexpressed in breast cancer. We performed a bioinformatic analysis using readily available online platforms to establish the prognostic and predictive effects related to CPT-1A expression. These include the KM plotter, the Human Protein Atlas, the cBioPortal, the G2O, the MethSurvand the ROC plotter. A Network analysis was performed using the Oncomine platform and signalling pathways constituting the cancer hallmarks, including immune regulation as utilised by NanoString. The epigenetic pathways were obtained from the EpiFactor website. Spearman correlations (r) to determine the relationship between CPT-1A and the immune response were obtained using the TISIDB portal. Overexpression of CPT-1A largely confers a worse prognosis and CPT-1A progressively recruits a range of pathways as breast cancer progresses. CPT-1A’s interactions with cancer pathways is far wider than previously realised and includes associations with epigenetic regulation and immune evasion pathways, as well as wild-type moderate to high penetrant genes involved in hereditary breast cancer. Although CPT-1A genomic alterations are detected in 9% of breast carcinomas, both the alteration and the metagene associated with it, confers a poor prognosis. CPT-1A expression can be utilised as a biomarker of disease progression and as a potential therapeutic target.

## Introduction

Breast cancer is the commonest malignancy in women and its incidence continues to rise with around 500,000 new cases each year according to the Global Cancer Observatory^1^. In 2020 the world health organisation (WHO) stated that the most commonly diagnosed cancer was breast with 2.26 million new cases^2^ whereas its incidence and mortality have been reported to be 46.8% and 13.6% respectively^1^. Breast cancer can be classified by its histological subtype, its receptor status or its molecular phenotype^3^. The mainstay of oncological management of breast cancer includes endocrine therapy, human epidermal growth factor receptor 2 (HER2) targeted therapy and chemotherapy. In clinical practise, the Nottingham Prognosis Index (NPI) is an index to determine prognosis following surgery for BC^4,5^, whereas a recent study using a multi-omics computational approach developed a prediction model for multi-class breast cancer NPI classes^6^. However, there is a need to identify further targets, particularly targets which can deal with the problem of tumour evolution and disease progression and that can be potentially used as therapeutic options.

It is well-established that global alterations in energy metabolism by cancer cells can promote tumourigenesis^7^. There is intense interest in probing metabolic pathways for biomarkers and therapeutic targets. While many metabolic pathways have received a lot of attention, lipid metabolism has been relatively under investigated but contains some promising targets^8^. Indeed, differences in expression of lipid metabolism related proteins in breast cancer subtypes has been demonstrated, with HER-2 positive tumours showing high expression, of PLIN1, CPT-1A, FASN, FABP4 and ACOX-1. Triple negative carcinomas, by contrast, showed low expression of PLIN1, CPT-1A and FABP4, and luminal. A cancers showed low expression of FABP4 and ACOX-1^9^. In addition, because the enzymes of oxidative metabolism are younger in evolutionary terms, there are fewer pathways available for tumour cells to evolve resistance to therapeutics targeting these enzymes; this makes oxidative metabolism a promising target to control tumour evolution.

The carnitine shuttle system represents the rate limiting step in fatty acid oxidation (FAO). A key component in this system is CPT-1 which resides on the outer mitochondrial membrane. It facilitates the formation of an acyl-carnitine complex which is then able to traverse the inner mitochondrial membrane via other members of the shuttle system and then eventually undergo beta oxidation^10^.

Three different isoforms of CPT-1 have been identified. CPT-1A appears to have the widest distribution in the body. It was originally considered as the hepatic isoform as its deficiency resulted in a rare genetic disorder of infants leading to hepatomegaly, seizures and sudden death. It has been found in the liver, pancreas, brain, kidney, blood, embryonic tissues, colon, duodenum and small intestine^11^. CPT-1B originally identified as the muscle isoform^12^ is expressed only in brown adipose tissue, muscle and heart and CPT-1C, known as the brain isoform^13^, is expressed in the brain and has also shown expression in testes. Overexpression of CPT-1A has been linked to progression of malignancy such as breast, prostate and lymphoma. It has also been shown that suppression of CPT-1 can lead to cell death and suppression of hallmarks of cancer progression^14^. Furthermore an integrated genomics approach has identified CPT-1A as a driver of proliferation in luminal breast cancers^15^ and it has also recently been suggested that CPT-1A could be used as a biomarker for disease monitoring in breast cancer^16^.

Drug therapies targeting CPT-1 have been developed for the treatment of heart disease and diabetes^17,18^. There has been increasing interest in targeting FAO in the context of cancer. The CPT-1 inhibitor etomoxir (ETO), which targets both CPT-1A and B, has been used in this context and has been shown to promote oxidative stress and impair cell proliferation in T cells^19^. ETO has also been shown to decrease proliferation without reducing cell viability in triple negative breast cancer cell lines that over express the oncogenic transcription factor MYC^20^. ST12326 also known as Teglicar; a newer, selective and reversible inhibitor of CPT-1A, has been shown to inhibit fatty acid oxidation in leukaemia cells with associated growth arrest, mitochondrial damage and apoptosis^21^. The results from these pre-clinical studies suggest a role for targeted inhibition of FAO in addition to current treatments available or as an alternative where others fail.

A splice variant of CPT-1A which is 11 amino acids shorter at the c-terminus, has been found to be expressed exclusively in the nuclei of the MCF7 (ER-positive) cell line when compared to the MCF12F cell line (non-tumorigenic epithelial mammary cells)^22^. Pucci et al. found that this variant, CPT-1Av2, forms a more stable complex with histone deacetylase-1 (HDAC1) and thus regulates genes involved in apoptosis, cell proliferation and invasion epigenetically^22,23^. HDAC1, in turn, has been implicated in mechanisms of immune evasion^24^.

Taken together, these data imply that CPT-1A has a wide range of interactions with pathways involved in a range of cancer hallmarks, mediated at least in part by epigenetic mechanisms. It was recently shown that CPT-1A is a marker of poor outcome in breast cancer^16^. Therefore, it should show clustering with one or more cancer hallmarks and the extent of these interactions has never been characterised in clinical cohorts. The aim of this analysis was to firstly characterise the predictive and/or prognostic value of CPT-1A as a biomarker at RNA and protein level using a range of online bioinformatics tools, and secondly to carry out a network analysis on the breast cancer patient cohorts available on the Oncomine platform^25^ to comprehensively characterise how CPT-1A clusters globally with genes involved in all known cancer hallmarks and epigenetic pathways.

## Methods

Assessment of the predictive and prognostic effect of CPT-1A expression at mRNA level was assessed using the KM plotter tool stratified by treatment and by molecular subtype^26^. The expression of CPT-1A was divided into high and low groups by splitting the mRNA expression level at the median. Kaplan Meier survival analysis was performed to assess the effect on overall survival.

The prognostic effect at protein level was assessed using the Human Protein Atlas^27^, in which CPT-1A expression was measured using immunohistochemistry and manually scored. Kaplan Meier survival analysis was performed to assess the effect on overall survival.

Alterations in the CPT-1A genome were assessed using the cBioPortal tool^28,29^, and the prognostic effect of this was assessed using the KM plotter and G2O tools^30^ using Kaplan Meier survival analysis.

The assessment of the prognostic effect of methylation of the CPT-1A gene was assessed using the MethSurv tool^31^ using Kaplan Meier analysis.

The effect of CPT-1A on treatment response was assessed using the ROC Plotter tool^32^ which used ROC analysis with complete pathological response and 5 year relapse free survival as short and long-term outcomes.

Network analysis was performed using the Oncomine platform^25^. The Oncomine platform has been discontinued since the completion of this study and is no longer available online. The extracted data used for this study is available in Supplementary Table 4. All other databases used in this study remain publicly available. The original data used in the Oncomine platform is publicly available from the TCGA website, the METABRIC website and the original publications.

The Oncomine platform has a built-in molecular concepts analysis tool. The tool automatically compares each gene set to each Oncomine cancer signature, assessing overlap significance with Fisher’s exact test. The Oncomine enrichment module then sorts gene sets of each type based on their degree of enrichment in a selected expression signature. Pre-defined lists of genes can be uploaded into Oncomine to be used in molecular concepts analysis. Molecular concepts analysis on these differential expression profiles identifies signalling pathways that are coordinately overexpressed^25^. The signalling pathways for the cancer hallmarks, including immune regulation, were based on the NanoString concepts^33^. The epigenetic signalling pathways were obtained from the EpiFactor website^34^. Eight breast cancer patient cohorts had data available on the Oncomine platform (Supplementary Table 3). Clustering of the signalling pathway with CPT-1A was taken as significant at a p < 0.01 and any odds ratio. The tool specified whether the clustering occurred in the context of over or under expression and specified the patient subgroup in which the clustering occurred. Subgroups identified in the platform included (i) subgroups related to stage, recurrence, grade and outcome, (ii) subgroups related to the histological subtype and receptor status of the tumour and (iii) other subgroups including, but not limited to, the presence of mutations, gender, smoking status and chemotherapy resistance. The results at signalling pathway level, and at individual gene level for genes involved in hereditary breast cancer, were summarised and presented with further detail in the supplementary information. The raw data specifies all of the genes in the concept which cluster with CPT-1A and are available in the Supplementary Materials.

Spearman correlations (r) to determine the relationship between CPT-1A and the immune response were obtained using the TISIDB portal^35^.

Correlation of CPT-1A with components of other metabolic pathways was obtained by calculating Pearson’s correlations of mRNA expression using the KM Plotter tool^26^.

## Results

### CPT1A expression at RNA level

**Figure 1:**
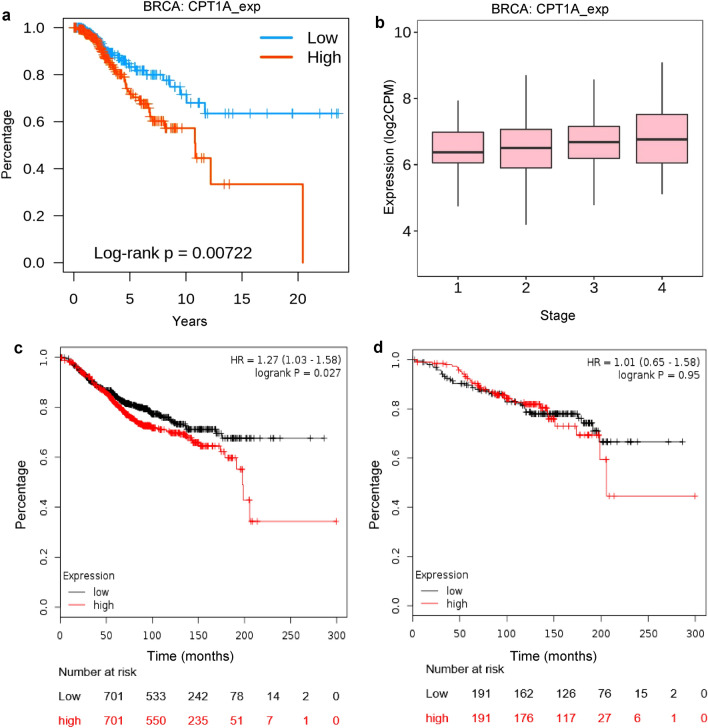
CPT-1A expression and clinical outcome in breast cancer. Kaplan–Meier graphs showing difference in overall survival of breast cancer patients with high and low expression of CPT-1A (**a**), correlation of CPT1A expression to disease stage depicted in box plot (**b**), spearman: rho = 0.089, p = 0.00355 and differences in overall survival of treated patients (**c**) vs. untreated patients (**d**).

Overexpression of CPT1A is a poor predictive marker at RNA level, confirmed on RNA expression data from both microarray and mRNA sequencing (Fig. 1, Table 1). High expression of CPT-1A in breast cancer patients correlates with a decreased overall survival (p = 0.007, Fig. 1a) and stage as more advanced breast cancers show higher expression of CPT-1A (p = 0.0035, Fig. 1b). Stratifying the patients by treatment status including all types of treatment showed that the CPT-1A predicted a poor outcome in treated patients only (p = 0.027, Fig. 1c,d).

**Table 1:** CPT-1A expression and breast cancer.

Molecular level	Source	Subgroup	Method	HR	P value	Outcome	n-number (low CPT-1A expression)	n-number (high CPT-1A expression)
mRNA	KM plotter (26)	Treated patients	RNA microarray	1.27	0.027	Poor	701	701
mRNA	KM plotter (26)	Patients treated with endocrine therapy	RNA microarray	1.15	0.62	NS	87	85
mRNA	KM plotter (26)	Patients treated with chemotherapy	RNA microarray	1.18	0.42	NS	200	198
mRNA	KM plotter (26)	Untreated patients	RNA microarray	1.01	0.95	NS	191	191
mRNA	KM plotter (26)	All breast carcinomas	RNA Seq	1.78	0.00042	Poor	591	498
mRNA	KM plotter (26)	Luminal A	RNA microarray	1.49	0.028	Poor	306	305
mRNA	KM plotter (26)	Luminal B	RNA microarray	1.51	0.028	Poor	216	217
mRNA	KM plotter (26)	HER2 positive	RNA microarray	1.01	0.98	NS	58	59
mRNA	KM plotter (26)	Basal phenotype	RNA microarray	1.15	0.57	NS	120	121
DNA alterations	KM plotter (26)	All breast carcinomas	DNA Seq	3.29	0.013	Poor	1089 (wild type)	8 (mutations)
mRNA metagene associated with mutation	G2O (30)	All breast carcinomas	DNA Seq and RNA microarray	0.76	8.6e−0.7	Poor	2024	2025
Protein	Human protein atlas (27)	All breast carcinoma	Immuno-histochemistry	1.80	0.00038	Poor	586	489

Summary table detailing hazard ratios (HR), p values and outcomes of overall survival data in breast carcinomas showing the predictive and prognostic effects of high CPT-1A expression at mRNA and protein level, and of CPT-1A mutations/alterations. The data has been obtained from the KM plotter, G2O and the Human Protein Atlas.

In addition, CPT-1A is a poor prognostic marker at protein level, confirmed on protein expression data from immunohistochemistry (p = 0.00038, Table 1). The effect at RNA level was preserved when patients were split by mutation burden and the enrichment or depletion of the immune compartments. When patients were split by molecular subtype, the predictive effect of CPT-1A was shown to occur in luminal A and luminal B breast cancers only (p = 0.028). There was no effect in HER2-positive or basal phenotype breast cancers (Table 1).

We also carried out an analysis using the ROC plotter tool^32^ to investigate whether CPT-1A was a biomarker of sensitivity or resistance to endocrine therapy, HER2-directed therapy or chemotherapy in breast cancers, both in terms of complete pathological response (CPR) and relapse-free survival (RFS). No significant effects were observed (Supplementary Figs. 1–10).

### Genomic alterations in CPT-1A

CPT-1A genomic alterations (mutations and copy number variations) and the metagene signature associated with CPT-1A alterations, both predicted a poor outcome (Table 1, Supplementary Fig. 11). Genomic alterations in the CPT-1A gene were detected in 9% of breast cancers in the cancer genome atlas (TCGA) and METABRIC cohorts (Supplementary Fig. 11). Alterations in CPT-1C, the brain isoform of CPT-1 and CPT-2 which sits on the inner mitochondrial membrane, are less frequent. The majority of the genomic alterations detected in all three genes were gene amplifications (Supplementary Fig. 11).

### Network analysis

**Figure 2:**
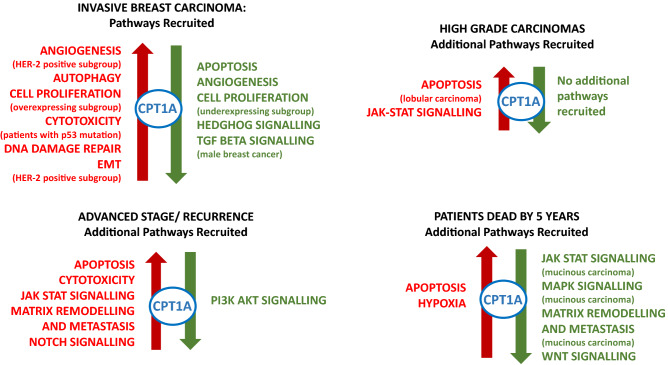
CPT1A clustering with cancer signalling pathways. Visual depiction of the clustering of CPT-1A with signalling pathways and whether the clustering occurs in the context of overexpression (red colour, left) or underexpression (green colour, right). The top left image shows the pathways which cluster with CPT-1A in the primary breast carcinomas. The remaining images show the additional pathways which cluster with CPT-1A in high grade carcinomas (top right), advanced stage or recurrence (bottom left) and in patients with a worse prognosis, as indicated by death by 5 years (bottom right). Where clustering is limited to a particular subgroup of carcinomas (defined by receptor status, histological subtype or the presence of p53 mutation), this is indicated in brackets beneath the signalling pathway.

Network analysis of the main cancer hallmarks and their associated pathways identified that CPT-1A clusters with genes in the autophagy, cell proliferation and DNA repair pathways in the context of gene overexpression (Fig. 2). There is also a subgroup of patients who show clustering of CPT-1A with cell proliferation in the context of underexpression. CPT-1A also clusters with genes in the angiogenesis and Hedgehog signalling pathways in the context of underexpression. High grade, poor survival and disease progression result in the association of CPT-1A with additional pathways, implying that CPT-1A recruits additional pathways as breast cancer progresses. Interestingly, the clustering of CPT-1A with JAK-STAT signalling, MAPK signalling, matrix remodelling and metastasis and Wnt signalling identifies a poor prognostic subgroup of mucinous carcinomas, a type of breast carcinoma which normally has a very good prognosis. A full breakdown of the analysis can be found in Supplementary Table 4.

In the individual gene level, CPT-1A showed clustering with wild type BRCA-1, BRCA-2, PIK3CA, ATM, TP53, PTEN, RAD51, EGFR and CDH1, all moderate to high-penetrant genes known to be mutated in hereditary breast cancer (Supplementary Table 1). In addition, CPT-1A clustered with cytotoxicity in breast tumours showing TP53 gene mutation (Supplementary Table 4).

### Epigenetic pathways

**Figure 3:**
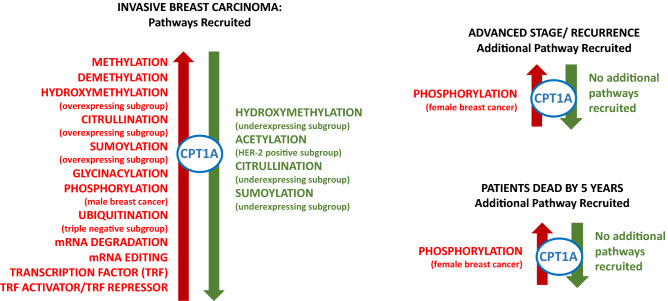
CPT1A clustering with epigenetic pathways. Visual depiction of the clustering of CPT-1A with signalling pathways and whether the clustering occurs in the context of overexpression (red colour, left) or underexpression (green colour, right). The remaining two images on the right show that phosphorylation is the only additional pathway to be recruited in female patients with advanced stage, recurrence and a worse prognosis, as indicated by death by 5 years. All male breast carcinomas showed clustering with phosphorylation in the primary tumours. Over and under-expressing subgroups were identified among the primary breast carcinomas for many of the signalling pathways.

CPT-1A clusters with all of the epigenetic pathways (Fig. 3). Most of the pathways are recruited in invasive carcinomas without evidence of progression, with the pathways of chromatin remodelling and histone phosphorylation being additionally recruited in the context of poor survival and advanced stage. Intriguingly, histone phosphorylation clusters across all carcinomas in male but not female breast cancer (Fig. 3). Methylation analysis of the CPT-1A identified that methylation of CPT-1A is detected in 45 loci, of which 23 are associated with a good prognosis (Supplementary Table 6).

### Immune pathways

**Figure 4:**
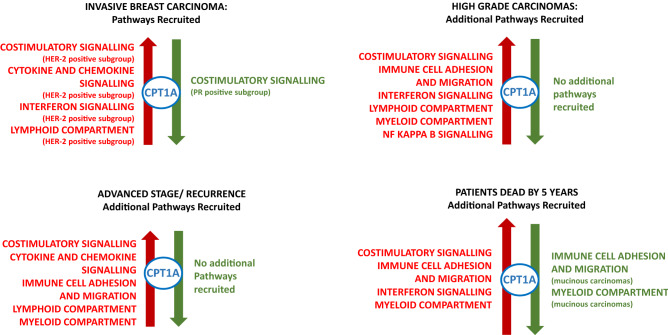
CPT1A clustering with immune pathways. Visual depiction of the clustering of CPT-1A with signalling pathways and whether the clustering occurs in the context of overexpression (red colour, left) or underexpression (green colour, right). The top left image shows the pathways which cluster with CPT-1A in the primary breast carcinomas. The remaining images show the additional pathways which cluster with CPT-1A in high grade carcinomas (top right), advanced stage or recurrence (bottom left) and in patients with a worse prognosis, as indicated by death by 5 years (bottom right). Where clustering is limited to a particular subgroup of carcinomas (receptor status or histological subtype), this is indicated in brackets beneath the signalling pathway.

CPT-1A shows clustering with all of the major immune pathways (Fig. 4). There is a general pattern for the clustering to occur in the context of gene overexpression in carcinomas overall and in the context of high tumour grade, poor survival and advanced stage. Clustering of CPT-1A with immune pathways in the context of underexpression occurs in tubular and mucinous carcinomas, both carcinomas which are associated with a good prognosis. Tubular carcinoma also shows clustering with the myeloid compartment in the context of overexpression (Fig. 4).

Increased expression of CPT-1A shows a negative correlation (r ≥ 1, p < 0.0001) with abundance of tumour infiltrating lymphocytes (TILs). The strongest correlations were observed with effector memory CD4 cells, T helper 1 cells (Th1) and Tγδ cells, CD56bright natural killer (NK) cells, NK T cells, activated dendritic cells (DCs) and plasmacytoid DCs. Methylation also showed a strong negative correlation with Tγδ and activated dendritic cells but a strong positive correlation to NK cells and eosinophils. The correlation data is analytically presented in Supplementary Table 2.

A negative correlation was identified between increased expression of CPT-1A and expression of immuno-inhibitory genes such as VTCN1/B7-H4 whereas a positive correlation was seen with KDR/VEGFR2 and IL10 expression. Increased methylation of CPT-1A was negatively associated with genes including IL10RB and positively associated with CD112 expression. No correlation was identified with the clinically relevant PD-L1 (CD274) and CTLA-4 genes (Supplementary Table 2).

### Metabolic pathways

Correlation with the components of the other metabolic pathways was weak overall (Supplementary Table 5). The two strongest correlations were a weak positive correlation with glucose transporters, and a weak inverse correlation with ceramide synthesis.

## Discussion

A recent single-centre study demonstrated that CPT-1A is a novel diagnostic and predictive biomarker for breast cancer^25^. In this study, utilising a bioinformatic approach, we showed that CPT-1A is a predictive and prognostic marker of a poor outcome in luminal A and luminal B breast cancers. The effect is confirmed in several different cohorts and by different methods at RNA and protein level, and also by the fact that CPT-1A identified poor prognostic subgroups in the network analysis. CPT-1A genomic alterations were detected in 9% of breast carcinomas, and are associated with a poor prognosis, as is the metagene associated with CPT-1A alterations. Moreover, methylation of CPT-1A, which would be expected to lower CPT-1A expression, is associated with a good outcome.

The network analysis revealed that CPT-1A clusters with genes involved in a wide range of cancer hallmarks, with a general pattern for CPT-1A to recruit more pathways as breast cancer progresses. The associations of CPT-1A with epigenetic and immune regulatory pathways are more extensive than was previously believed. There are also associations of CPT-1A with the wild-type variants of moderate to highly penetrant genes e.g. BRCA1, BRCA2 and ATM involved in hereditary breast cancer. Most of the associations were observed across all histological and molecular subtypes of breast cancer. In ER and PR-positive breast cancers, there was a tendency for CPT-1A to cluster in the context of under expression. Likewise, with the good prognostic histological subtypes, tubular and mucinous carcinomas, there was a tendency for CPT-1A to cluster in the context of under expression. A number of signalling pathways clustered with CPT-1A in HER2-positive breast carcinoma, although CPT-1A was not identified as a predictive or prognostic biomarker in this group. The reason for this discrepancy is not clear and warrants further investigation. The association of CPT-1A with these signalling pathways may be a consistent feature of HER2-positive breast cancer indicating the overall CPT-1A effect in cancer metabolism, conferring no additional predictive or prognostic effect in this group of patients.

In breast cancer cells, a shorter variant of CPT-1A, variant 2, has been identified in the MCF7 cell line^23^. This variant is located in the nucleus and has been shown to interact with HDAC1 forming a more stable complex illustrating a potentially important epigenetic role^23^ and identifies acetylation as the link between CPT-1A and epigenetic regulation. Our network analysis probed epigenetic links by identifying associations between CPT-1A and the genes involved in epigenetic regulation in patients. An association was found for CPT-1A with every epigenetic pathway we investigated. Unlike the signalling pathways of the cancer hallmarks, the epigenetic pathways were mostly recruited by CPT-1A across all carcinomas regardless of the state of progression of the tumour, with only histone phosphorylation and chromatin remodelling being associated with advanced disease. These associations suggest that the epigenetic regulatory network associated with CPT-1A is more extensive and complex than previously thought. Mechanistic studies in pre-clinical models will be needed to elucidate the interactions of the network and the functional role of CPT-1A within it.

The immune landscape of breast cancer is complex. Current understanding is that CD8^+^ T cells, CD4^+^ Th1 cells, NK cells, B cells, classically activated macrophages (M1) and mature dendritic cells are hostile to tumours and participate in tumour control and elimination. On the other hand, CD4^+^ Th2 cells, regulatory B cells, CD4^+^ T regulatory cells, myeloid-derived suppressor cells, and alternatively activated macrophages (M2) are co-opted by the tumour and support tumour development and progression^36^. The observation that increased CPT-1A expression and/or methylation negatively correlated with these cells suggests a possible mechanism for survival by suppressing the TILs response.

The co-option of immune cells in favour of tumour progression involves the recruitment of pro-inflammatory pathways including cytokine, chemokine, interferon and NF-kappa B signalling. It is therefore notable that CPT-1A clustered with genes in each of these signalling pathways, identifying groups with a poor prognosis or advanced disease. Clustering of CPT-1A in costimulatory signalling, the myeloid and lymphoid compartments, and immune cell adhesion and migration also identified subgroups with a poor prognosis or disease progression. These associations suggest that the effect of CPT-1A overexpression is being mediated by a tendency to promote the co-option of the immune system and to dampen tumour control. This is supported by the finding that clustering of CPT-1A with immune-related pathways in the context of under expression identified histological subtypes of breast carcinoma which are associated with a favourable prognosis. CPT-1A remained a poor prognostic marker when the components of the immune compartment were split into depleted and enriched subgroups. This indicates that the poor prognostic effect of CPT-1A is not solely dependent on immune mechanisms, a fact clearly borne out by the extent of the other associations seen. However, these data have revealed a previously unsuspected role for CPT-1A in the immune landscape of breast tumours. Recruitment of immune related pathways appears to be a late feature allowing the cancer to progress and to be an indicator of worse prognosis.

We have also observed a relationship between CPT-1A expression and expression of genes encoding for proteins targeted by immuno-inhibitors. Interestingly, a strong negative correlation is seen between CPT-1A expression and expression of VTCN1 which encodes for B7-H4; a membrane protein found on antigen presenting cells and an immune checkpoint molecule^36,37^. It has been shown that high expression of VTCN1 correlates with a response to tyrosine kinase inhibitors in HER2-positive breast cancer^38^. Increased CPT-1A expression could thus provide a mechanism of treatment resistance in these patients whereas silencing/inhibiting of CPT-1A will have a beneficial response to the patients. Further studies are needed to probe the mechanistic role CPT-1A plays in determining the balance of pro- and anti-tumour immune activity in the tumour microenvironment. It is certainly an avenue that is worth further exploration.

Studies in other cancer types support the role of CPT-1A as a driver of tumour progression^39,40^. Overexpression of CPT-1A confers a worse prognosis in several cancers^16,39,40^. In prostate cancer it has been shown that fatty acid oxidation plays an important role in cancer metabolism and that targeting CPT-1A with known inhibitors such as etomoxir leads to cell apoptosis and a reduction in cell proliferation^39^. It has also been shown that in colorectal cancer, the fatty acid oxidation pathway is activated in detached cells that are able to leave the primary tumour and that high levels of CPT-1A are seen in metastatic colorectal cells. Treatment of colorectal carcinoma cells with etomoxir prevented the formation of pulmonary and hepatic metastatic nodules in mice^40^. Due to its hepatotoxicity in phase II clinical trials the clinical use of etomoxir is limited^18^. Teglicar has been developed in the hope of achieving a better side effect profile and has been shown to inhibit fatty acid oxidation in leukaemia cells with associated growth arrest, mitochondrial damage and apoptosis^21^. Whilst a clinical use of these therapies is yet to be established, the results in vivo certainly give premise to further trials and studies targeting CPT-1A.

## Conclusions

This large scope analysis highlights further the potential of CPT-1A as a novel biomarker of breast cancer progression and to our knowledge, is the first study to demonstrate the associations of CPT-1A with immune regulation. Overall, overexpression of CPT-1A confers a worse prognosis. Broad epigenetic associations are established by the time the carcinoma has become invasive and, as the cancer progresses, more pathways involved in the cancer hallmarks and the immune pathways are recruited. Although associations with cell proliferation, apoptosis and invasion have been established in other cancer types and previous studies of breast cancer, the scope of the association of CPT-1A with other hallmarks including the immune pathways and epigenetic regulation, is a new and important observation that warrants further investigation. Therapeutics that target CPT-1A, originally developed to treat heart disease and diabetes, could be repurposed in the context of breast cancer as a way to achieve sustainable tumour control and as an adjunctive treatment to immunotherapies.

## Supplementary Information

Supplementary Figures.

Supplementary Table 1.

Supplementary Table 2.

Supplementary Table 3.

Supplementary Table 4.

Supplementary Table 5.

Supplementary Table 6.

## Data Availability

All data generated or analysed during this study are included in this article and its supplementary information files. The datasets supporting the conclusions of this article have been generated using the following websites: www.kmplotter.com, http://www.cbioportal.org; http://www.g-2-o.com; https://biit.cs.ut.ee/methsurv/; www.rocplotter.com; https://www.oncomine.org; https://epifactors.autosome.ru; http://cis.hku.hk/TISIDB/.

## Abbreviations

CPT-1: Carnitine palmitoyl-transferase-1
WHO: World health organisation
HER2: Human epidermal growth factor receptor 2
FAO: Fatty acid oxidation
ETO: Etomoxir
TN: Triple negative
PR: Progesterone receptor
ER: Estrogen receptor
HDAC1: Histone deacetylase-1
TILs: Tumour infiltrating lymphocytes
Th1: T helper 1
NK: Natural killer
DCs: Dendritic cells
TCGA: The cancer genome atlas
