# Supplementary material for: The role of CPT1A as a biomarker of breast cancer progression: a bioinformatic approach
Source: Sci Rep. 2022 Sep 30;12:16441. doi: 10.1038/s41598-022-20585-x (PMC9525709; doi:10.1038/s41598-022-20585-x)
Supplement: Supplementary file 1 — Supplementary Figures. [file 41598_2022_20585_MOESM1_ESM.pptx]

## Slide 1
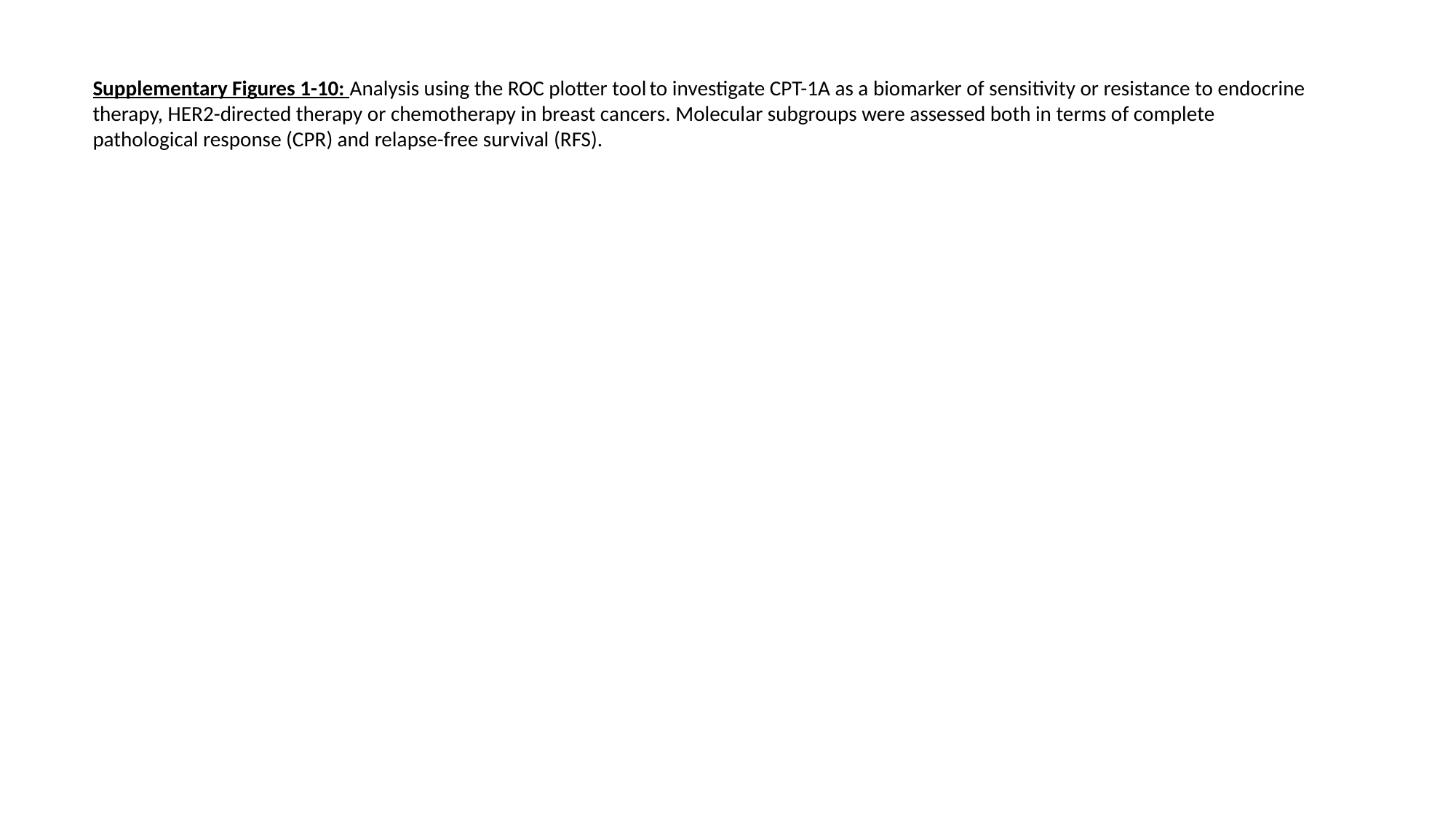

Supplementary Figures 1-10: Analysis using the ROC plotter tool to investigate CPT-1A as a biomarker of sensitivity or resistance to endocrine therapy, HER2-directed therapy or chemotherapy in breast cancers. Molecular subgroups were assessed both in terms of complete pathological response (CPR) and relapse-free survival (RFS).

## Slide 2
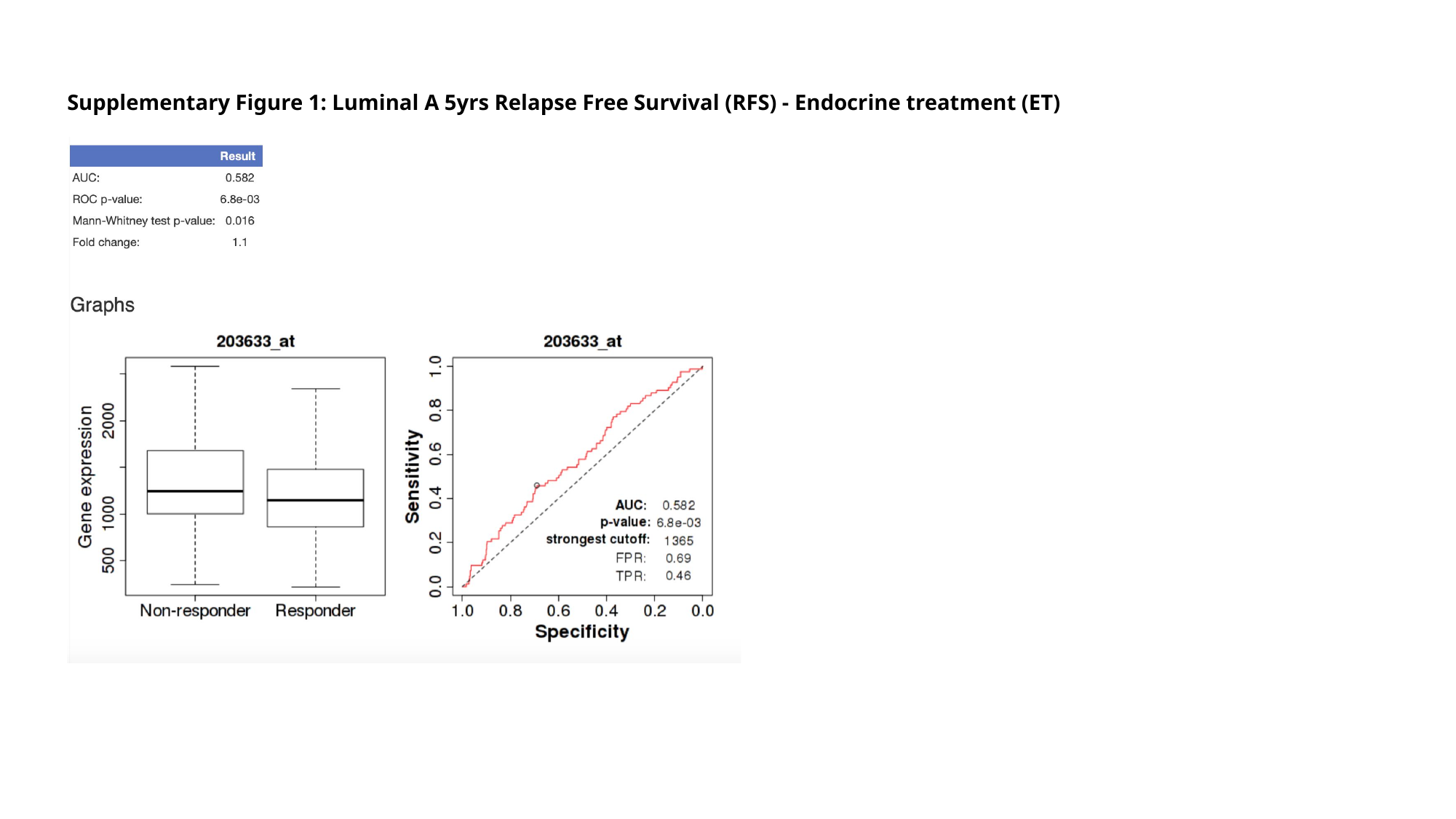

# Supplementary Figure 1: Luminal A 5yrs Relapse Free Survival (RFS) - Endocrine treatment (ET)

## Slide 3
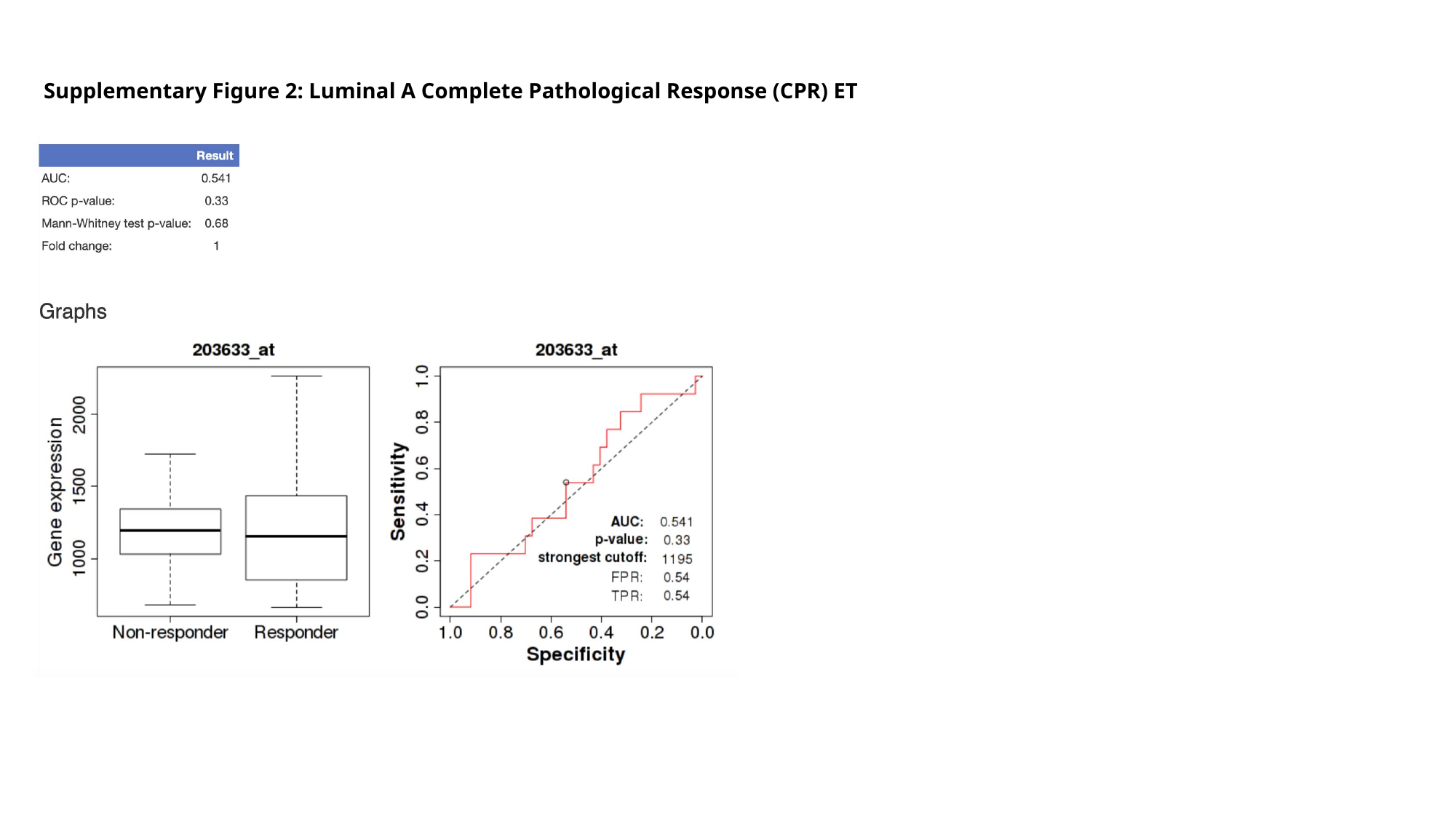

# Supplementary Figure 2: Luminal A Complete Pathological Response (CPR) ET

## Slide 4
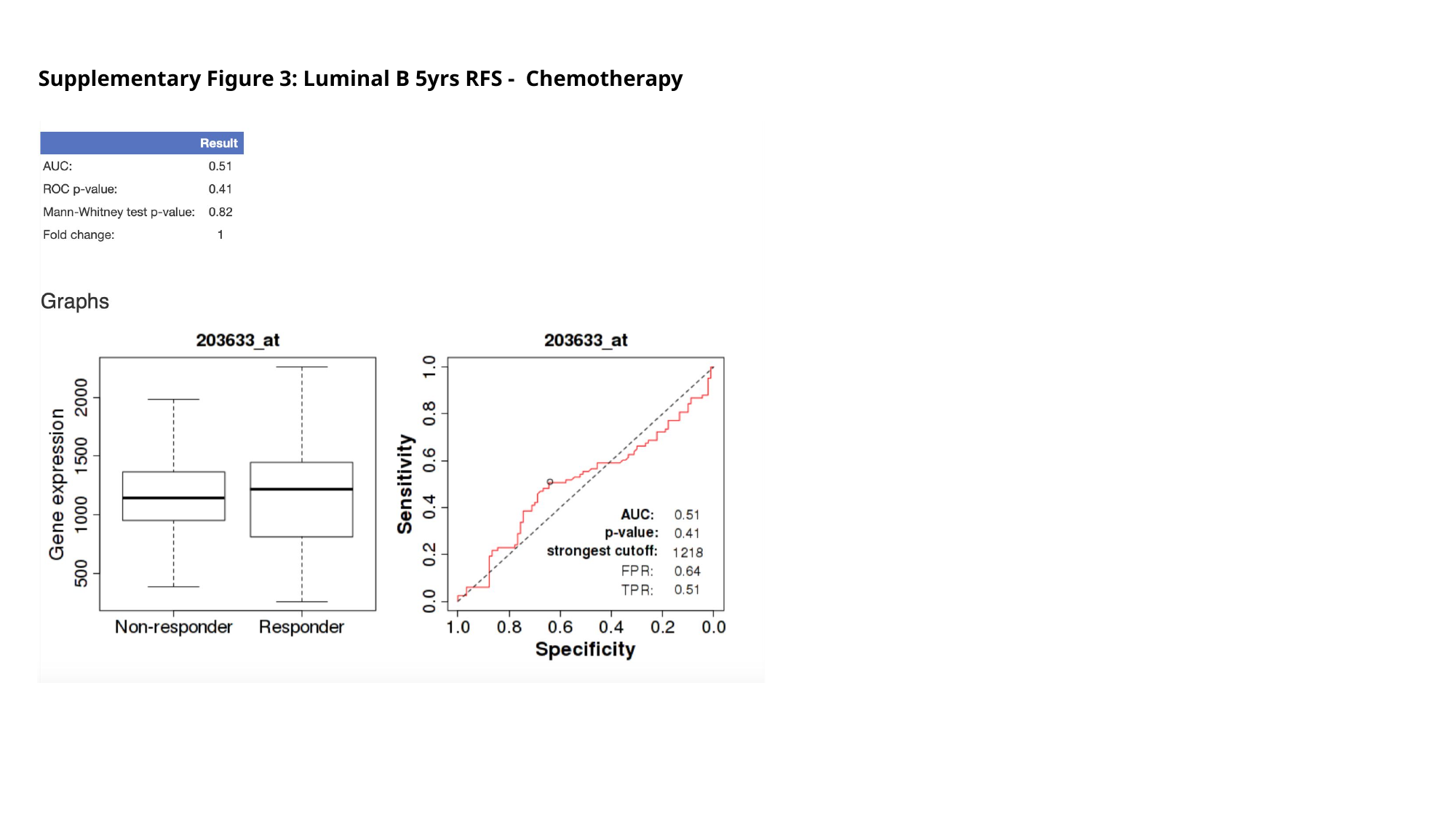

# Supplementary Figure 3: Luminal B 5yrs RFS - Chemotherapy

## Slide 5
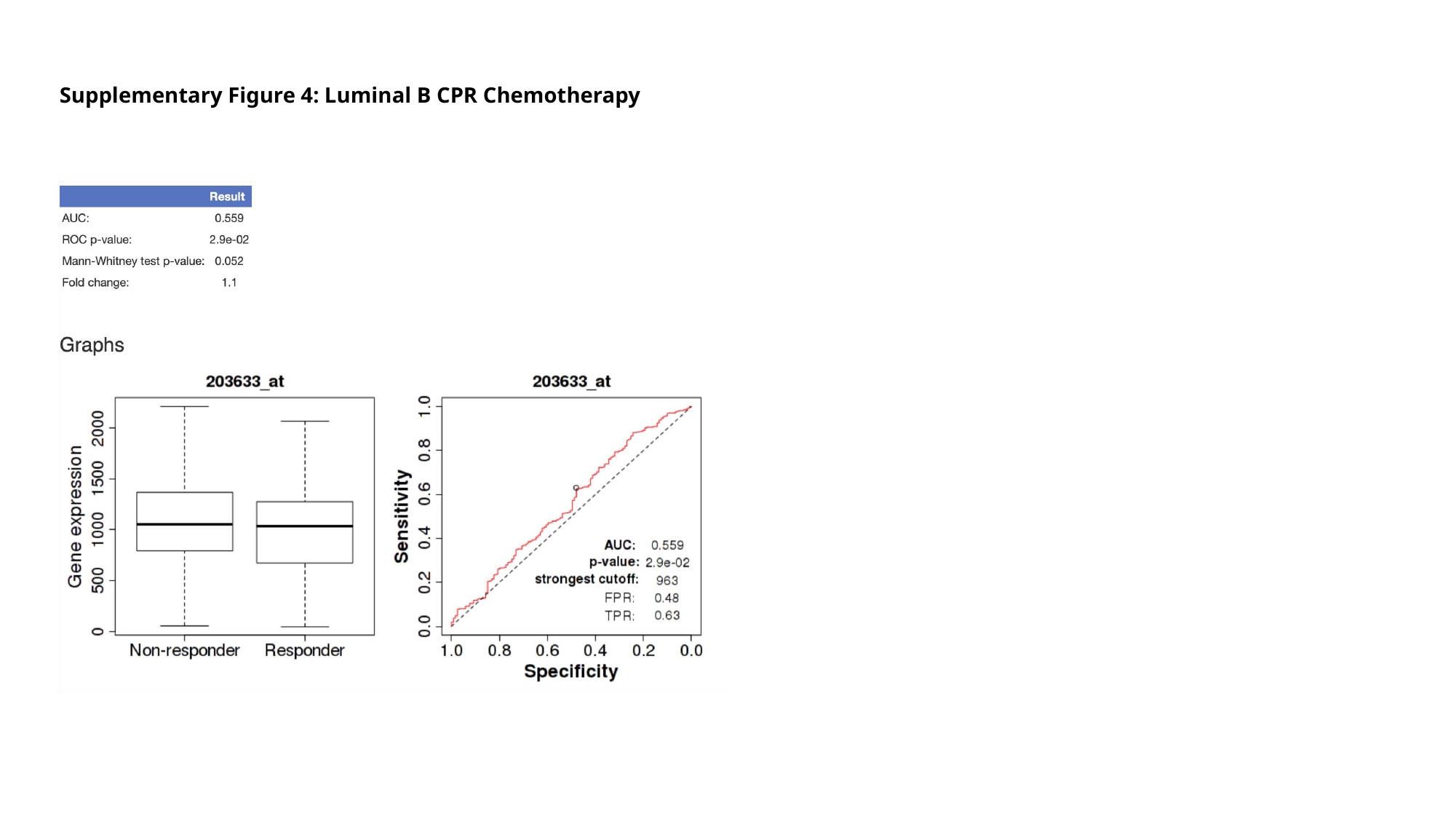

# Supplementary Figure 4: Luminal B CPR Chemotherapy

## Slide 6
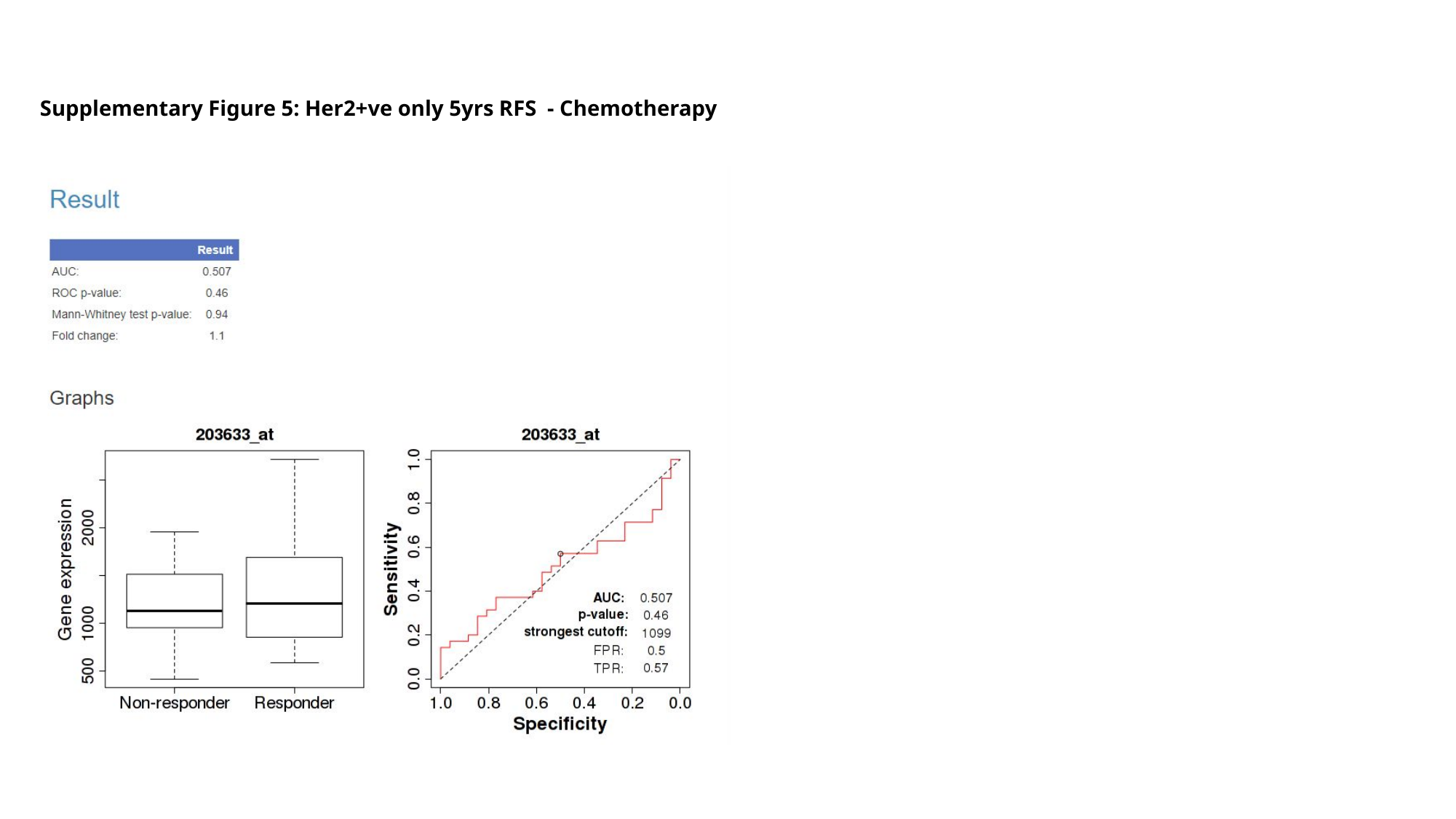

# Supplementary Figure 5: Her2+ve only 5yrs RFS - Chemotherapy

## Slide 7
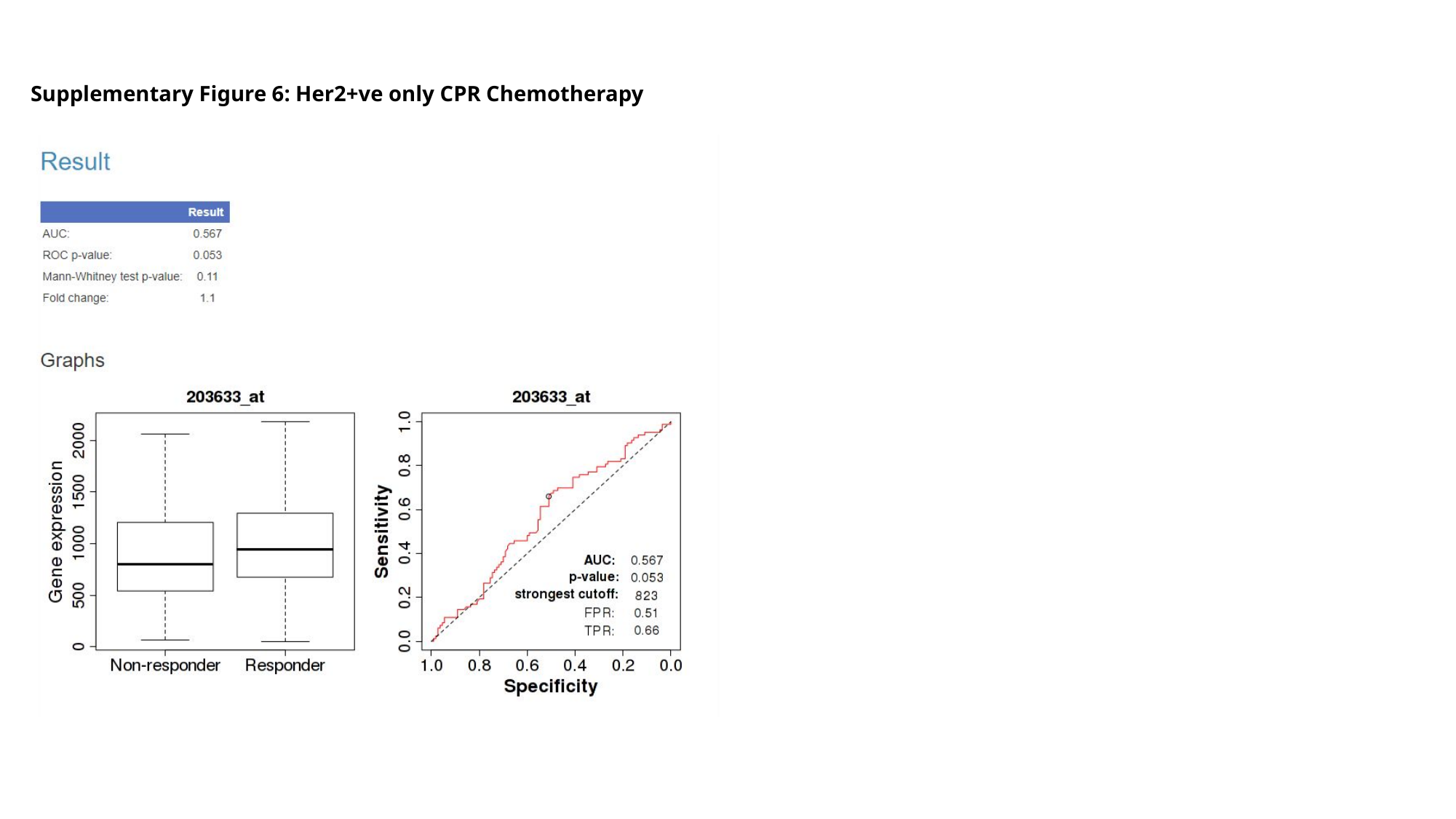

# Supplementary Figure 6: Her2+ve only CPR Chemotherapy

## Slide 8
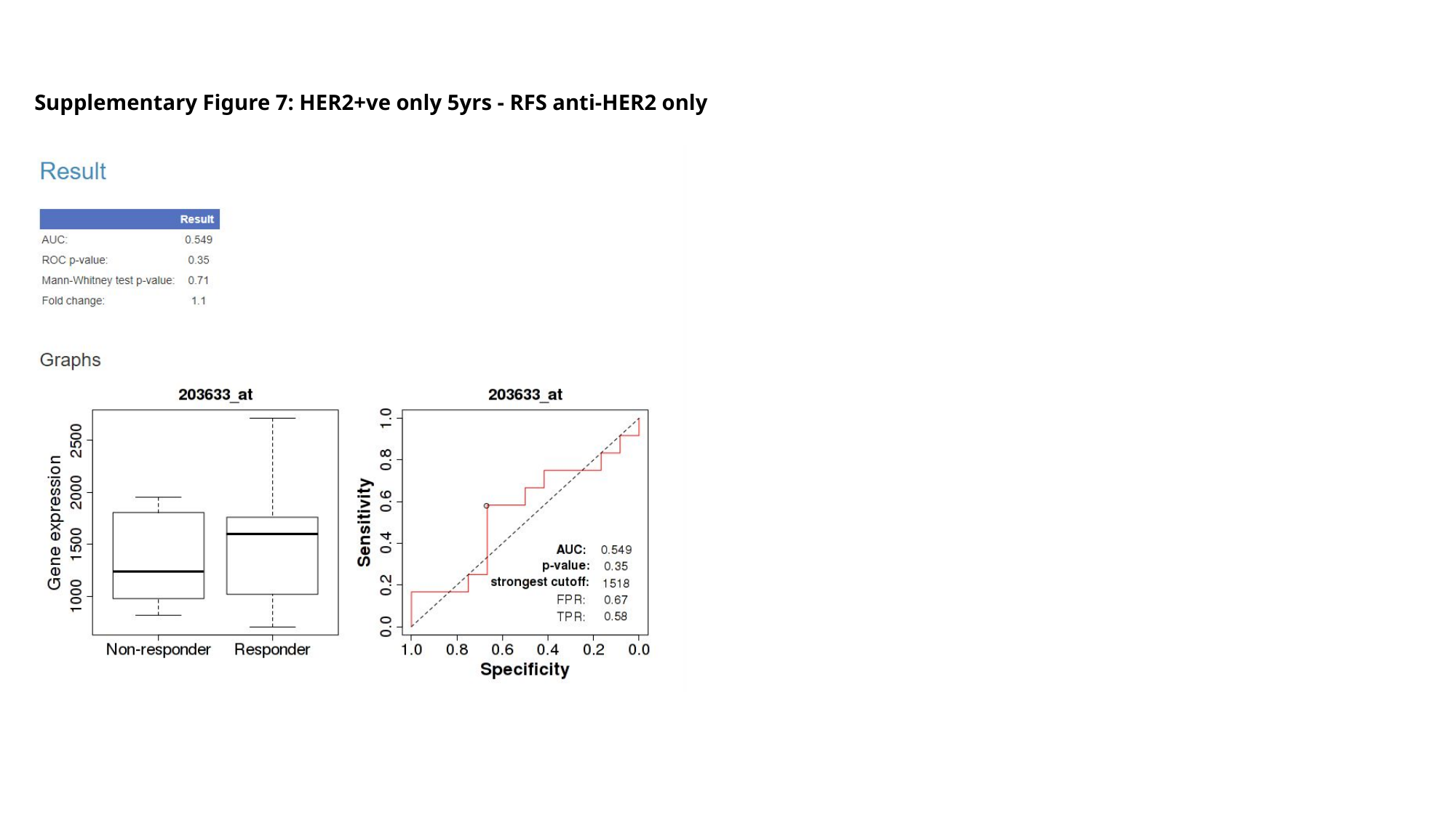

# Supplementary Figure 7: HER2+ve only 5yrs - RFS anti-HER2 only

## Slide 9
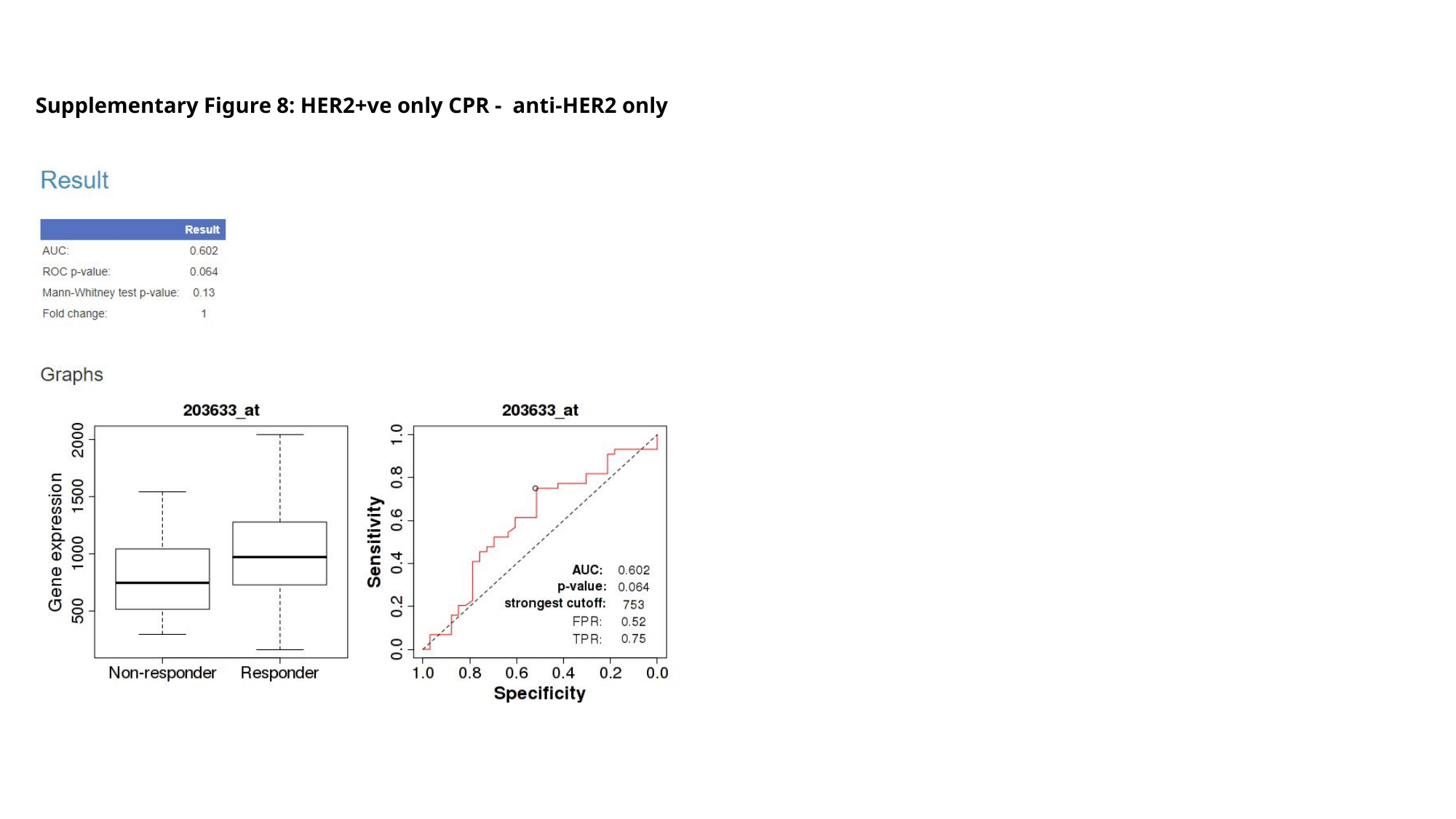

# Supplementary Figure 8: HER2+ve only CPR - anti-HER2 only

## Slide 10
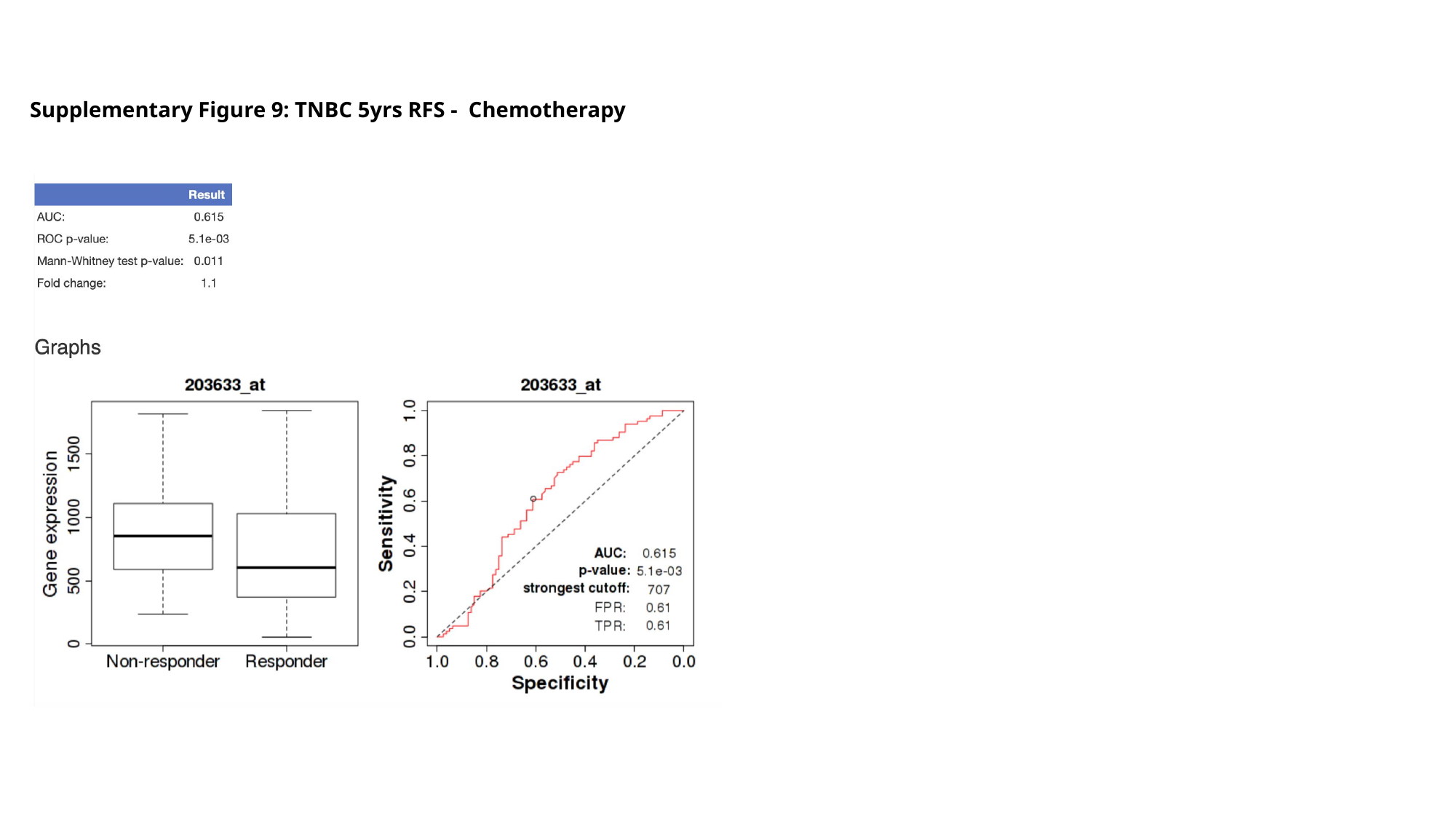

# Supplementary Figure 9: TNBC 5yrs RFS - Chemotherapy

## Slide 11
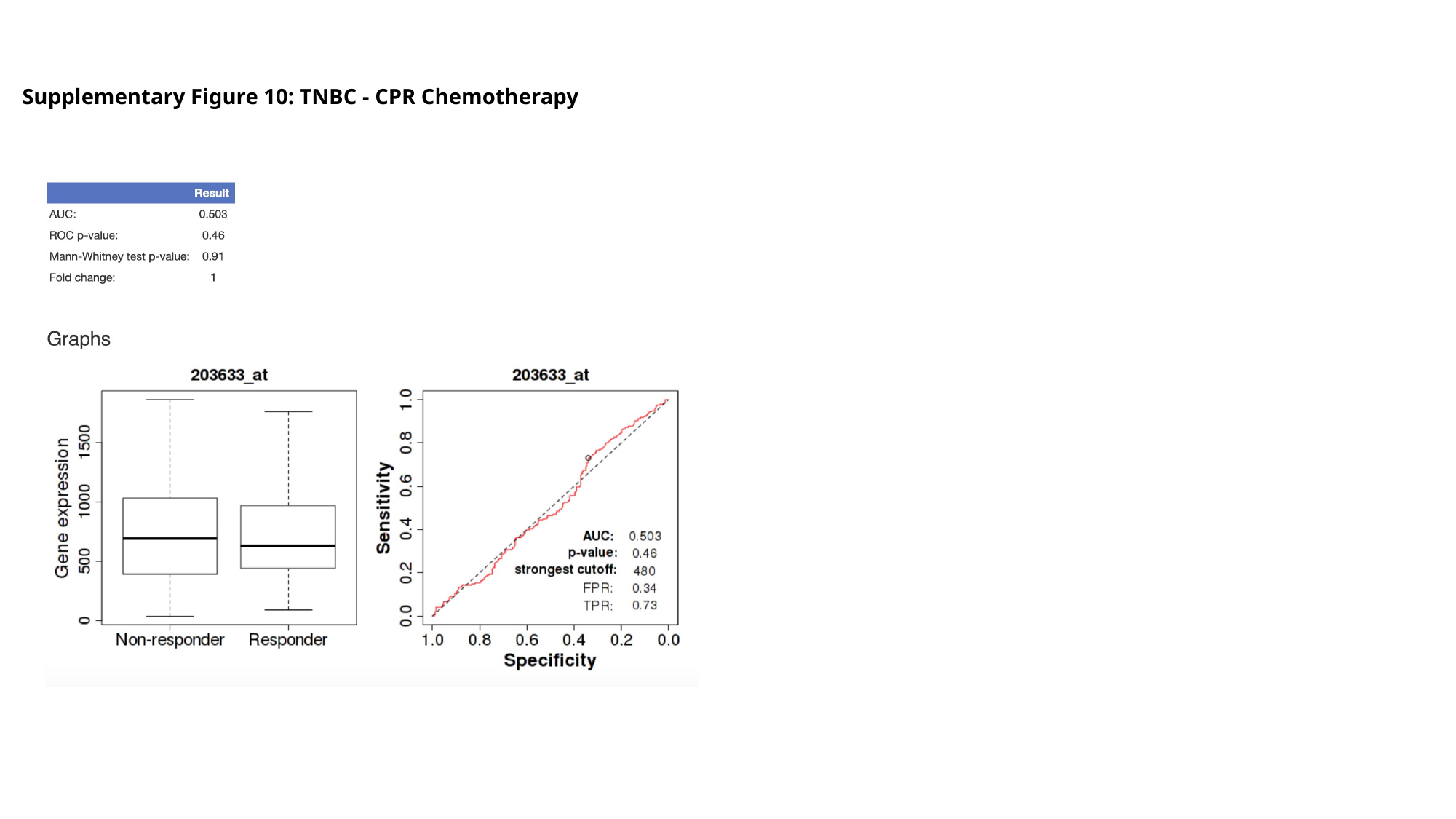

# Supplementary Figure 10: TNBC - CPR Chemotherapy

## Slide 12
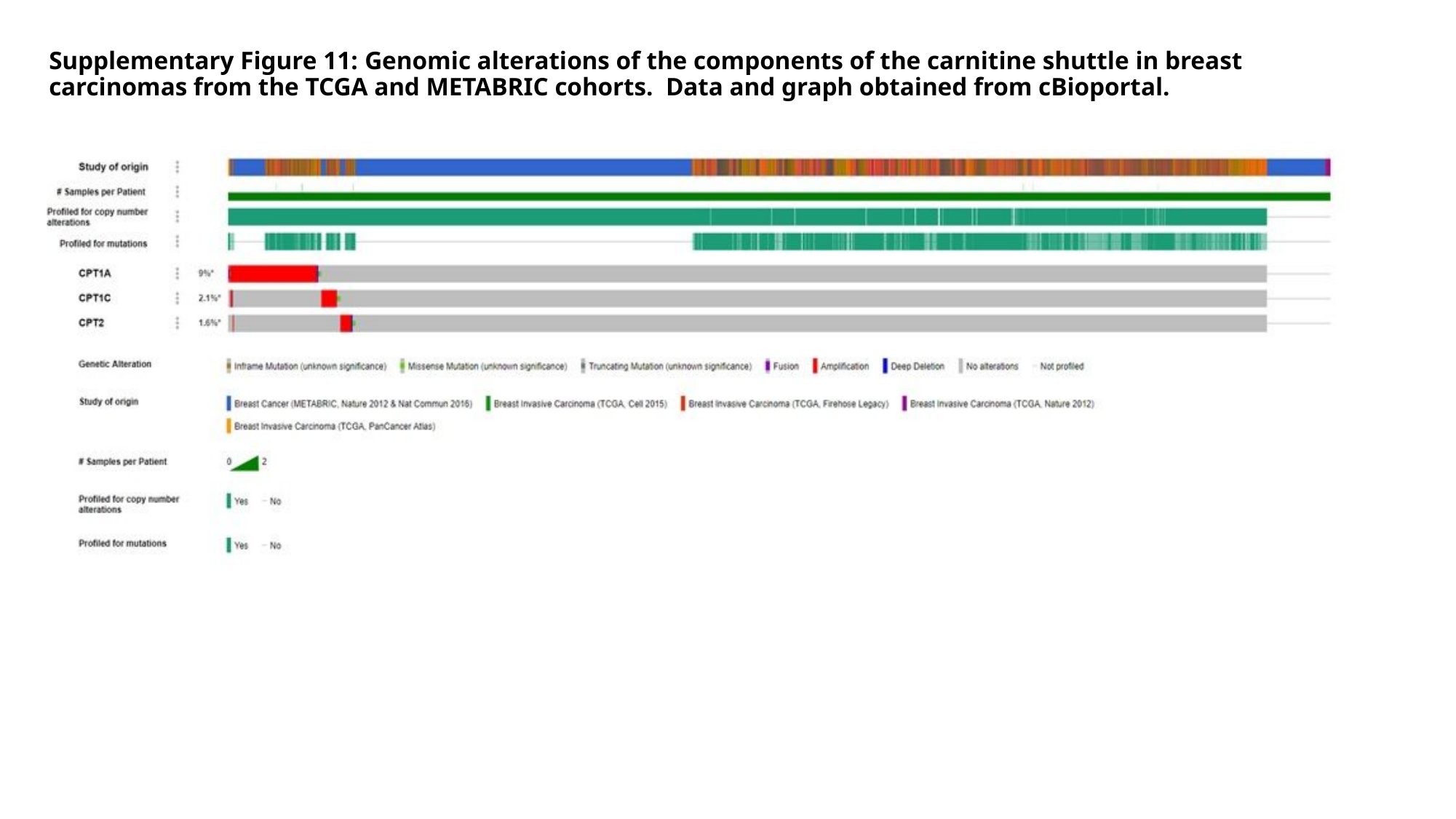

Supplementary Figure 11: Genomic alterations of the components of the carnitine shuttle in breast carcinomas from the TCGA and METABRIC cohorts. Data and graph obtained from cBioportal.
